# Supplementary material for: Transcriptomic Insights into Mechanisms of Early Seed Maturation in the Garden Pea (Pisum sativum L.)
Source: Cells. 2020 Mar 23;9(3):779. doi: 10.3390/cells9030779 (PMC7140803; doi:10.3390/cells9030779)

Zhongwan−6,25(1)

Global Summary

%DE = 0.19

genes with fdr < 0.2 = 398 (186 + / 212 −)

genes with fdr < 0.1 = 374 (178 + / 196 −)

genes with fdr < 0.05 = 335 (163 + / 172 −)

genes with fdr < 0.01 = 275 (139 + / 136 −)

genes in genesets = 0

<FC> = 0

<t−score> = 0

<p−value> = 0.01

<fdr> = 0.81

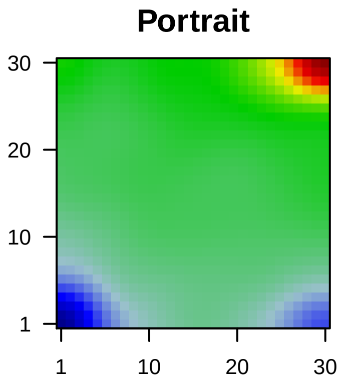

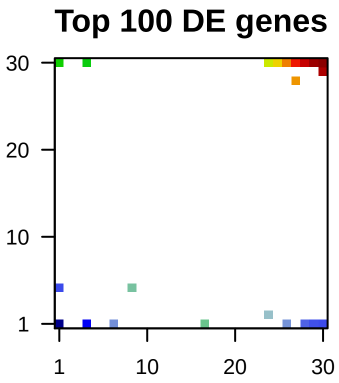

Global Genelist

Rank	ID	log(FC)	p−value	fdr	Metagene	Description
1	TRINITY_DN2	−0.52	2e−16	4e−15	1 x 1	
2	TRINITY_DN9	1.84	2e−16	4e−15	30 x 30	
3	TRINITY_DN7	0.97	2e−16	4e−15	30 x 1	
4	TRINITY_DN5	3.06	2e−16	4e−15	30 x 30	
5	TRINITY_DN5	0.59	2e−16	4e−15	1 x 1	
6	TRINITY_DN7	−1.21	2e−16	4e−15	1 x 1	
7	TRINITY_DN4	−3.25	2e−16	4e−15	1 x 1	
8	TRINITY_DN9	−0.73	2e−16	4e−15	30 x 1	
9	TRINITY_DN9	0.67	2e−16	4e−15	30 x 30	
10	TRINITY_DN2	0.64	2e−16	4e−15	29 x 30	
11	TRINITY_DN2	−0.71	2e−16	4e−15	1 x 1	
12	TRINITY_DN2	−0.78	2e−16	4e−15	30 x 1	
13	TRINITY_DN2	1.38	2e−16	4e−15	30 x 30	
14	TRINITY_DN2	−0.77	2e−16	4e−15	1 x 1	
15	TRINITY_DN3	−0.62	2e−16	4e−15	28 x 1	
16	TRINITY_DN3	−1.87	2e−16	4e−15	1 x 1	
17	TRINITY_DN3	−0.54	2e−16	4e−15	1 x 1	
18	TRINITY_DN1	−1.39	2e−16	4e−15	1 x 1	
19	TRINITY_DN2	−0.55	2e−16	4e−15	1 x 1	
20	TRINITY_DN8	1.17	2e−16	4e−15	30 x 30	

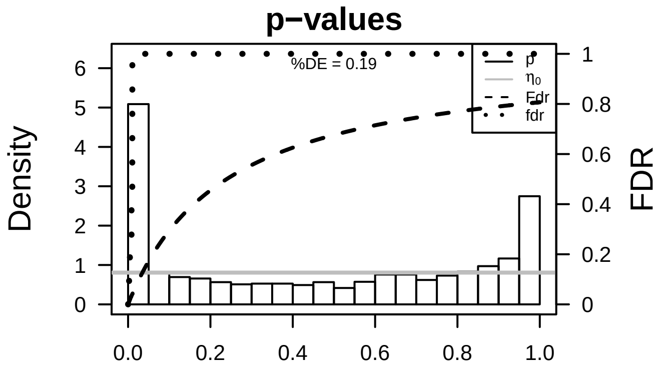

Supplement: Supplementary file 1 [file cells-09-00779-s001.zip › Supplementary materials/FileS4.zip!/FileS3/TFs - Results/Summary Sheets - Samples/Zhongwan.6.25.1..pdf]
